# TW-YOLO: An Innovative Blood Cell Detection Model Based on Multi-Scale Feature Fusion

**DOI:** 10.3390/s24196168

**Published:** 2024-09-24

**Authors:** Dingming Zhang, Yangcheng Bu, Qiaohong Chen, Shengbo Cai, Yichi Zhang

**Affiliations:** School of Computer Science and Technology, Zhejiang Sci-Tech University, Hangzhou 310018, China; 2021329621200@mails.zstu.edu.cn (D.Z.); 2021329621211@mails.zstu.edu.cn (Y.B.); 2021329621212@mails.zstu.edu.cn (S.C.); 2021329621037@mails.zstu.edu.cn (Y.Z.)

**Keywords:** YOLO, medical image, blood cell detection, multi-scale feature fusion

## Abstract

As deep learning technology has progressed, automated medical image analysis is becoming ever more crucial in clinical diagnosis. However, due to the diversity and complexity of blood cell images, traditional models still exhibit deficiencies in blood cell detection. To address blood cell detection, we developed the TW-YOLO approach, leveraging multi-scale feature fusion techniques. Firstly, traditional CNN (Convolutional Neural Network) convolution has poor recognition capabilities for certain blood cell features, so the RFAConv (Receptive Field Attention Convolution) module was incorporated into the backbone of the model to enhance its capacity to extract geometric characteristics from blood cells. At the same time, utilizing the feature pyramid architecture of YOLO (You Only Look Once), we enhanced the fusion of features at different scales by incorporating the CBAM (Convolutional Block Attention Module) in the detection head and the EMA (Efficient Multi-Scale Attention) module in the neck, thereby improving the recognition ability of blood cells. Additionally, to meet the specific needs of blood cell detection, we designed the PGI-Ghost (Programmable Gradient Information-Ghost) strategy to finely describe the gradient flow throughout the process of extracting features, further improving the model’s effectiveness. Experiments on blood cell detection datasets such as BloodCell-Detection-Dataset (BCD) reveal that TW-YOLO outperforms other models by 2%, demonstrating excellent performance in the task of blood cell detection. In addition to advancing blood cell image analysis research, this work offers strong technical support for future automated medical diagnostics.

## 1. Introduction

With the continuous development of deep learning, an increasing number of models are being applied to medical image processing, including the analysis of uterine ultrasound images [1], the diagnosis of glioblastoma [2], and the segmentation of bladder cancer images [3]. Blood cell detection represents a common small-scale object detection challenge in computer vision and a classic issue in medical imaging. Utilizing deep learning models for blood cell detection can significantly improve the accuracy and efficiency of detection, reducing human errors. It can automatically identify and classify different types of blood cells, promoting the advancement of medical diagnosis and research.

Using deep learning models for blood cell detection also faces multiple challenges. Firstly, blood cell images include various types of blood samples that have distinct differences in features and distribution compared to traditional imaging data. Blood cell images are more diverse, encompassing cells of different shapes, sizes, and colors. This diversity makes blood cell detection more challenging. Secondly, there is a significant size variation among cells in blood cell images. The images may contain large red blood cells as well as tiny platelets, and this scale difference increases the complexity of the detection task. Additionally, the background conditions in blood cell images are complex. Cells in the images may have similar textures and colors to the background, making them difficult to distinguish, further complicating the detection task. Moreover, images captured by microscopes may lead to detail loss and incomplete information, hindering precise cell detection. Finally, the quality of blood cell images can be affected by sample preparation, microscope settings, and shooting conditions. For instance, different microscope magnifications and lighting conditions can affect the quality and consistency of the images. These factors collectively contribute to the difficulties faced in object detection in blood cell images.

To tackle the challenges in blood cell detection and enhance detection capabilities, this paper presents the TW-YOLO model framework. We incorporate an RFAConv module to tackle the issues of cell morphological diversity and significant scale differences in blood cell detection tasks. Traditional CNNs (Convolutional Neural Network) have a fixed feature extraction structure, which leads to poor performance in extracting certain blood cell features. The RFAConv (Receptive Field Attention Convolution) structure significantly enhances the extraction of geometric features of blood cells. We also introduce the CBAM (Convolutional Block Attention Module) and EMA (Efficient Multi-Scale Attention) modules to significantly boost the model’s ability to extract features. Additionally, an innovative PGI-Ghost (Programmable Gradient Information-Ghost) method is proposed by us to better describe feature extraction and gradient flow in blood cell detection, consequently enhancing the performance of the model.

The main contributions of this paper are as follows:

1. This study presents an innovative TW-YOLO framework for blood cell detection and target recognition.
2. By introducing the RFAConv, the model’s feature extraction capabilities have been enhanced. The introduction of the EMA algorithm allows the model to better focus on areas of interest. The addition of the PGI module makes the gradient flow more stable during the convergence process, while the inclusion of the GhostModule increases the model’s detection speed.
3. Experiments on blood cell image datasets demonstrate that this approach can significantly boost the recognition capability of the YOLO model in blood cell detection.

## 2. Related Work

### 2.1. YOLO

CNN has achieved significant milestones in the development of deep learning, and YOLO is a model of great importance within CNN. Owing to its straightforward design, efficient network, and outstanding detection capabilities, the YOLO model is extensively employed in the realm of object detection. Yang et al. [4] achieved good results in forest fire smoke detection by improving the detection head of the YOLO algorithm. Yu et al. [5] made significant progress in detecting and analyzing corrosion on coated metal surfaces by enhancing the YOLOv5 algorithm. Wang et al. [6] offered an innovative BPN-YOLO algorithm to boost the model’s performance in wood defect detection. To better achieve immature yellow peach target detection, Xu et al. [7] presented the EMA-YOLO model, enhancing the framework’s proficiency in identifying small-scale targets. Liu et al. [8] designed the YOLO-GP algorithm based on YOLOv8, achieving good results in hazardous behavior detection. Yang et al. [9] integrated the ECA attention mechanism into the bottleneck layer and proposed the FE-YOLO model, which improved the detection accuracy of construction waste in complex scenarios. Wang et al. [10] applied the YOLOv8 model for rapeseed flower counting.

In most methods, the effectiveness of the YOLO framework is often strengthened by adding attention mechanisms and other techniques. However, in deep learning, these methods tend to encounter the problem of gradient vanishing as the model depth increases. To better alleviate this issue, we propose the PGI-Ghost strategy to mitigate information loss and bottleneck problems in deep neural network models, generating reliable gradients.

### 2.2. Application of Deep Learning Methods in Medical Image Processing

In recent years, the notable advances in deep learning have led to an increasing number of these models being applied to medical image processing. Wang et al. [11] applied hyperparameter reconstruction and the YOLO model to colorectal polyp detection, achieving excellent results. Balasubramani et al. [12] used the YOLO model for left ventricle segmentation in echocardiography. Vahdati et al. [13] applied deep learning models to thyroid nodule detection in ultrasound imaging, providing good assistance in diagnosis. Madankumar et al. [14] presented a novel retinal vessel segmentation model based on RF-UNet. Ramaekers et al. [15] used deep learning models to reduce the difficulty of detecting and locating pancreatic cancer in CT scans. Toosi et al. [16] used deep learning to predict non-segmentation results for head and neck cancer PET/CT images, reducing the need for manual delineation of malignant tumors on FDG PET-CT images. Cheng et al. [17] utilized GANs to capture virtual contrast in realistic contrast-enhanced MRI imaging, thereby reducing the risk associated with the use of contrast agents.

For the task of blood cell detection, this paper introduces the RFAConv and EMA modules to address the rich geometric shapes and diverse scales of blood cells. These modules effectively boost the model’s capacity for extracting features, boosting the performance of the model in blood cell detection.

### 2.3. Blood Cell Detection

The detection of blood cells is a classic task in computer vision and medical imaging. The main challenges lie in the complex geometry of blood cells and the small size of the available datasets. Chen et al. [18] proposed the MFDS-DETR model by designing methods such as an advanced feature fusion pyramid, which improved the model’s performance in blood cell detection. Raghaw et al. [19] proposed the CoTCoNet model, which integrates dual feature extraction to capture long-range global features and fine-grained spatial patterns, enhancing the model’s ability to extract features from blood cells. Liu et al. [20] highlighted that the detection of white blood cells is challenging due to their varying shapes and staining levels in complex real-world scenarios. To address this issue, they proposed a white blood cell detection method based on dual feature fusion, called TFF CenterNet. Wang et al. [21] proposed a method that combines Fourier ptychographic microscopy with YOLO, enabling the acquisition of high-resolution, wide-field blood cell images in a single shot, thereby improving detection accuracy. K. Gokulkannan et al. [22] designed a multi-scale adaptive and attention-based DCNN (MAA-DCNN) approach and developed a new multi-scale Trans-Res-Unet3+ (MTResUnet3+) model using this method, enhancing the detection performance for pathological blood cells.

These models have improved performance through model fusion; however, they often come with high computational complexity and lack good real-time capabilities. We proposed the TW-YOLO model based on the YOLO framework, which enhances detection accuracy while maintaining the YOLO model’s strong real-time performance.

## 3. Proposed Method

**Figure 1:**
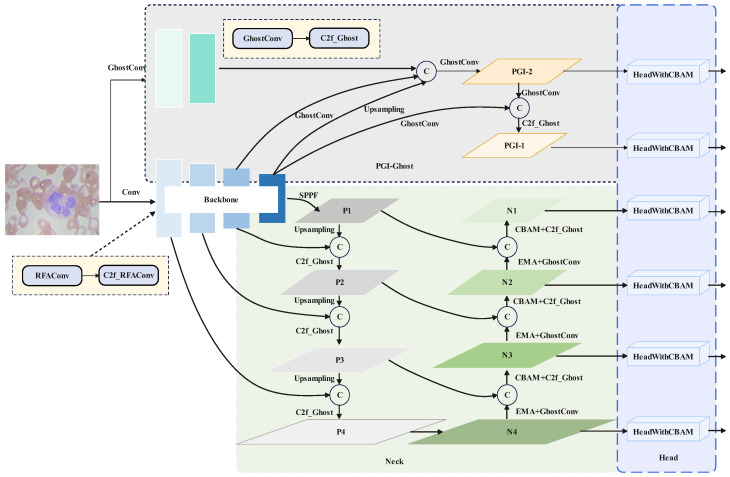
This image shows the framework of TW-YOLO. In the Backbone, there are several RFAConv and C2f_RFAConv structures. The Neck part includes the GhostModule, EMA mechanism, and CBAM module. PGI-Ghost provides better gradient flow. Finally, there are six detection heads with CBAM modules that output the final results.

Based on the YOLOv8s framework, this work proposes the TW-YOLO model. In the TW-YOLO framework, RFAConv, CBAM, and EMA modules are introduced based on the characteristics of the blood cell detection task to strengthen the capacity of the model in extracting features. The PGI-Ghost strategy is proposed to allow the model to form richer gradient flows and provide better supervision to the backbone network. At the same time, the GhostModule is introduced to decrease the model’s computational demands. TW-YOLO improves the feature representation capability and strengthens the performance in blood cell detection tasks. Figure 1 shows the TW-YOLO architecture.

The TW-YOLO algorithm is mainly composed of the Backbone, Neck, PGI-Ghost, and Head. Before inputting into the model, TW-YOLO adaptively adjusts the resolution of the input image to 640×640. Before training start, we use Mosaic data augmentation to combine four pictures through randomly cutting, brightness adjustment, flipping, and other methods. The Backbone extracts features from the images in the framework. The part of Neck adopts the framework of the Feature Pyramid Network (FPN) [23] and Path Aggregation Network (PAN) [24]. The feature pyramid uses upsampling to transfer strong semantic information from deep feature maps to shallow layers. PGI-Ghost directly connects different levels of the auxiliary network to the backbone network, forming richer gradient flows. The Path Aggregation Network uses downsampling to transfer shallow positional information to deeper layers for multi-scale feature fusion. Finally, the decoupled detection head with the CBAM attention mechanism outputs the detection boxes, confidence scores, and classes.

It is worth mentioning that we use two attention mechanisms in the model, each with different tasks. CBAM, after the initial multi-scale feature fusion, refines these fused features more precisely. Through its dual attention mechanism on the channel and spatial dimensions, it further optimizes feature representation, enabling the network to focus more accurately on important features and locations. EMA, on the other hand, is better suited for capturing the fine details of these refined features. It extracts features across multiple scales, allowing the model to better integrate local and global information, thereby enhancing the model’s ability to perceive details at different scales.

In terms of multi-scale feature fusion, compared to YOLOv8, we added an extra small-scale feature fusion layer in the neck, allowing the model to better detect small-scale blood cells. Additionally, we introduced a PGI-Ghost feature fusion layer to integrate features with the backbone, providing better gradient flow. In the detection head, we added a small-scale detection head directly connected to the small-scale feature fusion layer, while two other detection heads are connected to the PGI feature fusion layer, enabling the model to achieve improved detection results.

### 3.1. RFAConv

**Figure 2:**
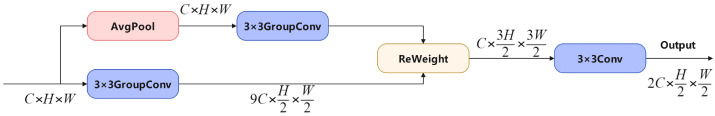
This picture illustrates the RFAConv computation process.

In traditional CNNs, the output of each convolution operation is based on a small window of the input data, also known as a local receptive field. However, in blood cell detection, due to the shape of blood cells, not every feature in a window is equally important. Therefore, to better extract blood cell features, we introduced RFAConv [25] in TW-YOLO. The specific computation process of RFAConv is shown in Figure 2.

RFAConv performs AvgPool on the input features. Following global pooling, a GroupConv process is applied to the feature map. At the same time, GroupConv process is also performed for the input features. The feature maps resulting from the two GroupConv operations are then subjected to ReWeight operation. Finally, the final output result is obtained after another convolution. The formula for AvgPool is shown in Equation (1):

(1) $$Y_{i,j}=\frac{1}{k\times k}\sum_{m=0}^{k-1}\sum_{n=0}^{k-1}F_{i+m,j+n}$$

where $F_{i+m,j+n}$ represents the input feature map value at position, $Y_{i,j}$ is the output feature map value at position i,j, and *k* denotes the pooling kernel size. The equation describes the average of the values within a k×k window of the input feature map is computed to reduce the spatial dimensions while preserving essential information.

By using RFAConv, the network can produce unique weights for each receptive field. This means that the convolution kernel can dynamically adjust its parameters based on the varying features in each receptive field, rather than applying the same processing to all areas. RFAConv evaluates the significance of each location inside the receptive field through a spatial attention mechanism and adjusts the weights of the convolution kernel accordingly. Therefore, each receptive field has a unique convolution kernel rather than utilizing a common kernel for all receptive fields. This approach enables the framework to more finely learn local features within the image, thereby enhancing the overall network performance. By using this method, RFAConv enhances the model’s representation capability, allowing it to more accurately adapt to and represent the features of the input data, especially when dealing with complex or variable image content.

**Figure 3:**
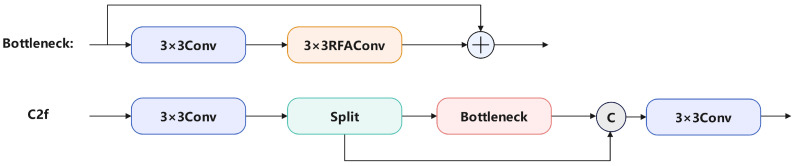
The computation process of C2f integrating RFAConv.

To further enhance the model’s feature extraction effectiveness, we modified the YOLOv8’s C2f structure by integrating RFAConv. The modified C2f structure is shown in Figure 3. We incorporated RFAConv into the bottleneck, improving the feature fusion capability within the C2f structure.

Through the RFAConv module, the model is more capable to extract features from targets, allowing TW-YOLO to perform better in blood cell detection.

### 3.2. Convolutional Block Attention Module

In YOLOv8, the model uses the Concat operation to fuse features from two scales. This operation is simple and efficient, but in blood cell detection, where blood cells have diverse shapes and rich scale features, a simple Concat operation does not achieve satisfactory results. Therefore, the Convolutional Block Attention Module (CBAM) [26] was used within the detection head and feature fusion layer.

**Figure 4:**
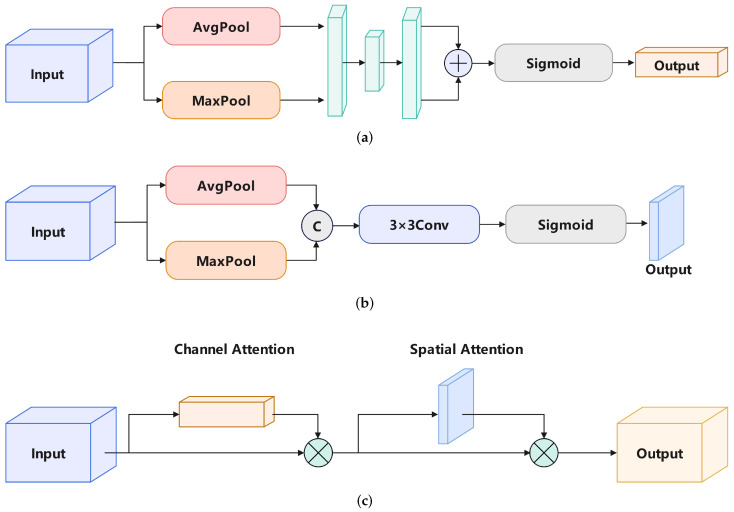
The CBAM module and its attention mechanism. (**a**) Channel Attention; (**b**) Spatial Attention; (**c**) CBAM.

CBAM has two important attention mechanisms: Channel Attention and Spatial Attention. Figure 4a shows computation process of Channel Attention. The input feature map undergoes separate AvgPool and MaxPool processes. The calculation method for AvgPool is shown in Equation (1), and the calculation method for MaxPool is shown in Equation (2):

(2) $$Y_{i,j}={Max}_{0\leq m<k,0\leq n<k}F_{i+m,j+n}$$

where $F_{i+m,j+n}$ represents the input feature map value at position, $Y_{i,j}$ is the output feature map value at position i,j, and *k* denotes the pooling kernel size. The equation defines the MaxPool operation, where the maximum value within a k×k window of the input feature map is selected as the output.

The results of the two pooling operations are then added after passing through two same fully connected layers. The final result will be obtained through a Sigmoid activation function. The sigmoid is calculated as shown in Equation (3):

(3) $$Sigmoid\left(x\right)=\frac{1}{1+e^{-x}}$$

where *x* represents the input value to the Sigmoid function. The Sigmoid function is used to map the input values to a range bet ween 0 and 1, which is essential for generating attention maps that assign importance to different features within the model.

Figure 4b shows the computation process of Spatial Attention. Once AvgPool and MaxPool processes are applied to the input feature map, the results are merged, followed by a convolution and a Sigmoid activation function to produce the last result. The CBAM’s computation result is shown in Figure 4c. To achieve the final output, the input features are sequentially multiplied by the Channel Attention and Spatial Attention mechanisms.

To enable TW-YOLO to achieve better feature fusion, we integrated the CBAM structure after the Concat operation, allowing the model to have improved multi-scale feature fusion capability. By incorporating CBAM, the model can more accurately focus on important feature areas, which is especially beneficial for blood cell detection tasks. Additionally, to get better results in the detection head, we incorporated a CBAM module into the detection head to further improve the accuracy of bounding box localization.

**Figure 5:**
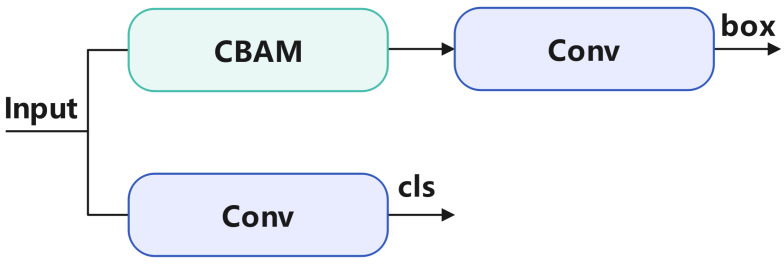
This figure illustrates the decoupled detection head integrated with the CBAM in the TW-YOLO model. This structure enhances the model’s ability to accurately localize bounding boxes by applying attention mechanisms to improve feature prioritization after multi-scale feature fusion.

The framework of the integrated detection head is shown in Figure 5. The YOLOv8 model uses a decoupled detection head, where the input features are processed through two separate convolutional layers to output the detection box coordinates and object categories. We introduced the CBAM module before the bounding box coordinate output, allowing the model to more accurately localize the bounding boxes.

Specifically, after the Concat operation, which fuses features from different scales, the combined features are split into two paths. One path passes through the CBAM module, where both channel and spatial attention mechanisms are applied. These mechanisms help the model prioritize significant features, particularly when detecting complex and variably shaped blood cells. The optimized features are then processed by a convolutional layer before entering the bounding box decoder, which outputs the detection box coordinates. The other path goes through a separate convolutional layer to output the object classes.

TW-YOLO boosts the model’s ability to fuse features by incorporating the Concat operation and decoupled detection head with CBAM, enabling better feature extraction for blood cells.

### 3.3. Ghost Module

**Figure 6:**
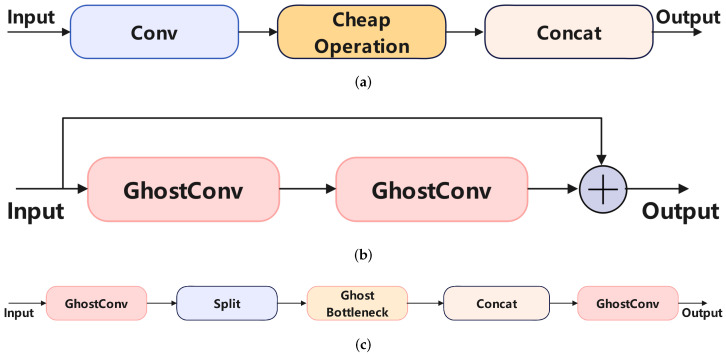
This figure shows the GhostConv and GhostC2f modules used in the TW-YOLO model. These modules reduce computational complexity by applying a lightweight convolution process, which maintains accuracy while improving efficiency in feature extraction. (**a**) GhostConv; (**b**) Ghostbackbone; (**c**) GhostC2f.

In traditional convolutional neural networks, the extensive use of convolutions leads to significant computational costs. To decrease the model’s computational expenses, we introduced the Ghost Module [27] into TW-YOLO. Figure 6 illustrates the GhostConv and GhostC2f modules integrated into the TW-YOLO model. GhostConv performs a lightweight convolution by first applying convolution to half of the input channels and then using a simple linear transformation to generate the remaining features, significantly reducing computational complexity without sacrificing accuracy. GhostC2f further integrates this GhostConv mechanism into the C2f structure, optimizing feature extraction while maintaining model efficiency in blood cell detection tasks. This approach allows TW-YOLO to achieve a balance between performance and computational cost.

Figure 6a shows the computation process of GhostConv. For the input feature map, a convolution is initial performed with half the channels, followed by a simple linear transformation. Finally, the results of the convolution and the linear transformation are concatenated together. This computation method reduces computational complexity without sacrificing accuracy. As shown in Figure 6b,c, we introduced GhostConv into the C2f structure, reducing the computational complexity of C2f.

By incorporating GhostConv, the complexity of TW-YOLO is reduced without decreasing accuracy, resulting in better performance in blood cell detection.

### 3.4. Efficient Multi-Scale Attention

**Figure 7:**
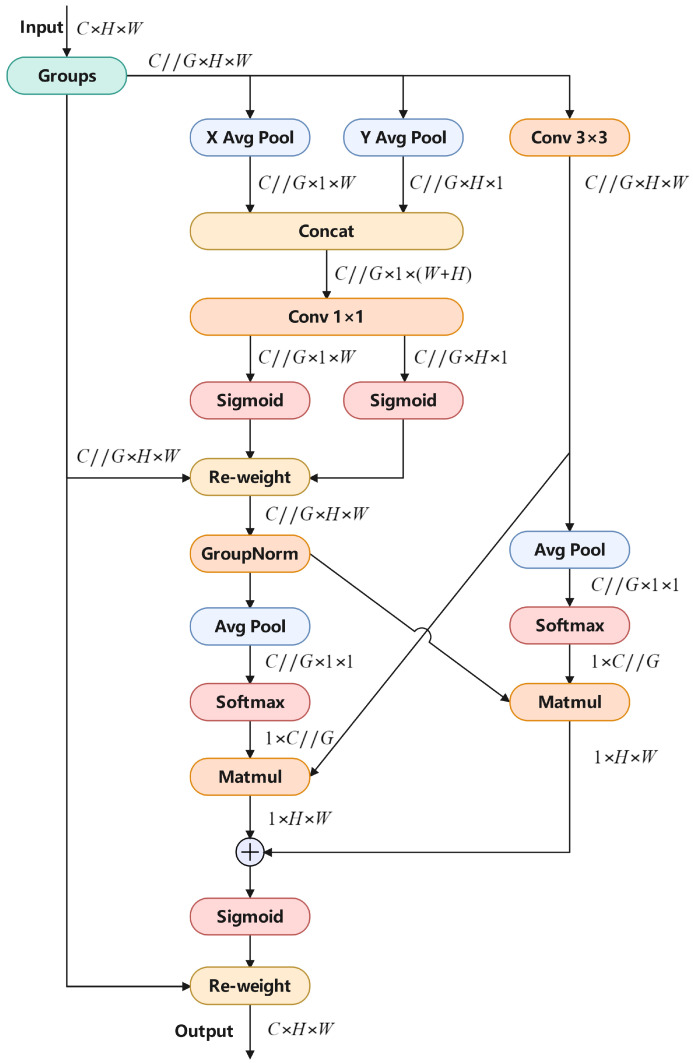
This figure shows the EMA module’s process, which uses average pooling in horizontal and vertical directions to capture important spatial features. This enhances TW-YOLO’s ability to detect details across different scales in blood cell images.

In blood cell detection, we face the challenge of diverse blood cell geometries. To better overcome this difficulty, we incorporated the Efficient Multi-Scale Attention (EMA) module [28]. Figure 7 shows the EMA computation process. Figure 7 depicts the computation process of the Efficient Multi-Scale Attention (EMA) module in the TW-YOLO model. The input features are divided into groups and undergo average pooling along both the horizontal and vertical dimensions, capturing important spatial information at different scales. The pooled features are then concatenated, passed through a convolutional layer, and combined with the original features using a Softmax function. This process enhances the model’s ability to focus on key regions of interest, improving feature extraction and detection accuracy, particularly in complex blood cell images.

We assume that the form of the input features in the EMA module is C×H×W. First, the features are divided into *G* groups. Each group’s feature shape is C//G×H×W. For the features of each group, X avg pool and Y avg pool are performed separately. The formula for X avg pool is shown in Equation (4):

(4) $$Y_{c}^{W}=\frac{1}{W}\sum_{0\leq i\leq W}F_{c}\left(H,i\right)$$

where $Y_{c}^{W}$ represents the output feature map after applying average pooling along the width dimension, $F_{c}\left(H,i\right)$ is the input feature map for channel *c*, and *W* is the total width of the feature map. X avg pool strengthens the focus on the spatial regions of interest in the horizontal direction through average pooling along the horizontal dimension. The formula for Y avg pool is shown in Equation (5):

(5) $$Y_{c}^{H}=\frac{1}{H}\sum_{0\leq i\leq W}F_{c}\left(i,W\right)$$

where $Y_{c}^{H}$ represents the output feature map after applying average pooling along the height dimension, $F_{c}\left(H,i\right)$ is the input feature map for channel *c*, and *W* is the total width of the feature map. Y avg pool strengthens the focus on the spatial regions of interest in the vertical direction through average pooling along the vertical dimension.

The EMA model concatenates the features processed by X avg pool and Y avg pool, followed by a 1×1 convolution. The resulting features are then split and subjected to an activation function, after which procedure is conducted using the original features. Global pooling is then applied to the Re-Weighted feature map. Meanwhile, the original feature map undergoes a 3×3 onvolution followed by global pooling. Afterward, it passes through a Softmax function, as shown in Equation (6):

(6) $$Softmax\left(x_{i}\right)=\frac{e^{x_{i}}}{\sum_{j=1}^{n}e^{x_{j}}}$$

where $x_{i}$ is the *i*-th element of the input feature and *n* is the dimension of the input feature. This normalization ensures that the output values are scaled between 0 and 1, with the sum of all output values equaling 1.

The feature maps generated before and after applying global pooling on both sides are first multiplied element-wise. After this multiplication, the resulting feature maps are then added together to produce the final output features.

The EMA module enhances the model’s focus on the spatial regions of interest in both horizontal and vertical directions, thus boosting the model’s effectiveness for detect blood cells applications. The EMA specific location in TW-YOLO is shown in Figure 1.

### 3.5. Programmable Gradient Information-Ghost

In traditional FPN and PAN, features at different levels are continuously concatenated, convolved, and fused, eventually passed to the detection head to obtain the loss. This results in indirect gradient propagation during model training, causing the deep network to lack supervision and leading to information loss. To address this issue, YOLOv9 [29] first proposed the Programmable Gradient Information (PGI) strategy, forming an auxiliary network with unidirectional convolution, ensuring direct gradient flow in the auxiliary network during backpropagation. Furthermore, by using multi-level gradient fusion, different levels of the auxiliary network are directly connected to the backbone network, ensuring that even with complex concatenation loops, the backbone network can form reliable gradients. Additionally, the auxiliary network provides reliable supervision to address the problem of information loss in deep networks. In this paper, we improve the original PGI strategy and propose Programmable Gradient Information-Ghost (PGI-Ghost). This algorithm is applied in our more complex feature fusion network, where the Ghost module is used to lightweight the auxiliary network, reducing parameters while forming richer gradient flows and providing better supervision for the backbone network. Additionally, the auxiliary network in the PGI-Ghost strategy can be removed during model export, significantly enhancing inference speed.

## 4. Experiment

### 4.1. Experiment Settings

The original picture resolution is resized to 640×640 during preprocessing in this research. The Adam optimizer is selected for parameter optimization, with an initial learning rate of 0.01 and a weight decay coefficient of 0.0005. The model is trained for a total of 150 epochs, with a bias learning multiplier set to 0.1 and a learning rate momentum of 0.937. We use Mosaic data augmentation [30] to enhance the dataset, where four images are randomly scaled, cropped, rotated, and then stitched together. All training and testing are conducted on the NVIDIA RTX 4090 using Pytorch 2.0.1 and CUDA 11.8. To achieve better training results, the BatchSize is set to 32.

### 4.2. Datasets

To better validate the capability of TW-YOLO in blood cell detection, we introduced four blood cell detection datasets for experiments. The four datasets are: BloodCell-Detection-Dataset (BCD) [31], Complete Blood Count (CBC) [32], BCCD [33], and LISC [34]. BCD, CBC, and BCCD are three datasets that categorize blood cells into three classes: WBC, RBC, and Platelets. The BCCD dataset contains 364 photos with a resolution of 640×480 pixels. The CBC dataset consists of 360 blood smear photos, all having a resolution of 640×480 pixels. The BCD dataset has 801 pictures with the resolution of 416×416 pixels. The LISC dataset contains a total of 250 images, with all cells classified into five categories: neutrophils (NEU), eosinophils (EOS), monocytes (MON), basophils (BAS), and lymphocytes (LYM). To better test and validate the performance of TW-YOLO in blood cell detection, we split the training and testing sets in an 8:2 ratio.

### 4.3. Evaluation Metrics

To evaluate the model’s effectiveness on the dataset, we utilize mAP50 as the evaluation metric. Before calculating mAP50, we need to compute the IoU of the detection box. Assuming detection box A is the box predicted by framework and B is the ground real box, the computation process for IoU is illustrated in Equation (7):

(7) $$IoU=\frac{A⋂B}{A⋃B}$$

For mAP50, if the IoU between the model’s predicted box and the ground truth box is greater than 0.5, the detection box is considered correct. Based on this, we need to calculate the Precision for each category. The formula is shown in Equation (8):

(8) $$Precision=\frac{TP}{TP+FP}$$

where TP denotes the total amount of correctly predicted targets and FP signifies the total amount of detection boxes predicted by the model that are incorrect. The final calculation method for mAP50 is shown in Equation (9):

(9) $$mAP=\frac{1}{m}\int_{1}^{m}AP$$

To evaluate the computational complexity and detection speed of the model, we used the Giga Floating Point Operations per Second (GFLOPs) metric and Frames Per Second (FPS) metric. The parameter metric refers to the number of trainable variables, such as weights and biases, in a machine learning model. It is significant because models with more parameters generally have higher capacity to learn complex patterns, but they also require more computational resources and may risk overfitting if not properly regularized. GFLOPs is a measure of a model’s computational complexity. It represents the number of billions of floating-point operations a model performs per second. Lower GFLOPs usually indicate a faster, more efficient model, while higher GFLOPs suggest greater computational demands. FPS is a measure of how many images or frames a model can process in one second. It is commonly used to assess the speed and real-time performance of a model, with higher FPS values indicating faster processing and better real-time capabilities.

### 4.4. Experimental Results

We conducted a comparison research about the proposed model versus other methods. Outcomes of the comparison are displayed in Table 1. Table 1 shows the mAP50 for each category as well as the overall mAP50. As illustrated in Table 1, TW-YOLO achieved mAP50 scores of 0.947, 0.962, and 0.967 on the BCCD, BCD, and CBC datasets, respectively, showing a significant advantage over other models. On the BCCD dataset, TW-YOLO improved the mAP50 by 0.042, 0.024, 0.051, 0.02, 0.04, and 0.034 compared to YOLOv8s, YOLOv5x, YOLOv7, and CST-YOLO, respectively. On the BCD dataset, TW-YOLO improved the mAP50 by 0.017, 0.007, 0.021, 0.006, 0.014, and 0.008 compared to YOLOv8s, YOLOv5x, YOLOv7, CST-YOLO, YOLOv9s, and YOLOv10s, respectively. On the CBC dataset, TW-YOLO showed even more significant improvements, with mAP50 increases of 0.02, 0.083, 0.089, 0.056, 0.034, and 0.027 compared to YOLOv8s, YOLOv5x, YOLOv7, CST-YOLO, YOLOv9s, and YOLOv10s, respectively. The above findings indicate that TW-YOLO not only more precisely identifies more targets compared to other models, but also demonstrates better recognition capabilities for different types and complexities of targets when processing the BCCD, BCD, and CBC blood cell detection datasets. Particularly in terms of the mAP50 metric, TW-YOLO shows a clear advantage across all datasets, demonstrating its superior accuracy in target detection and recognition, highlighting the advantages and potential applications of the TW-YOLO model in the field of blood cell detection.

Table 2 shows the performance of various models on the LISC dataset. Table 2 shows the mAP50 for each category as well as the overall mAP50. Although the TW-YOLO model has slightly lower accuracy in recognizing certain categories compared to other models, it achieves improvements in overall mAP50, outperforming YOLOv8s, DETR, YOLOv5-ALT, YOLOv9s, and YOLOv10s by 0.012, 0.005, 0.006, 0.019, and 0.009, respectively. These results demonstrate TW-YOLO’s excellent feature extraction capabilities and robustness in blood cell detection tasks.

**Figure 8:**
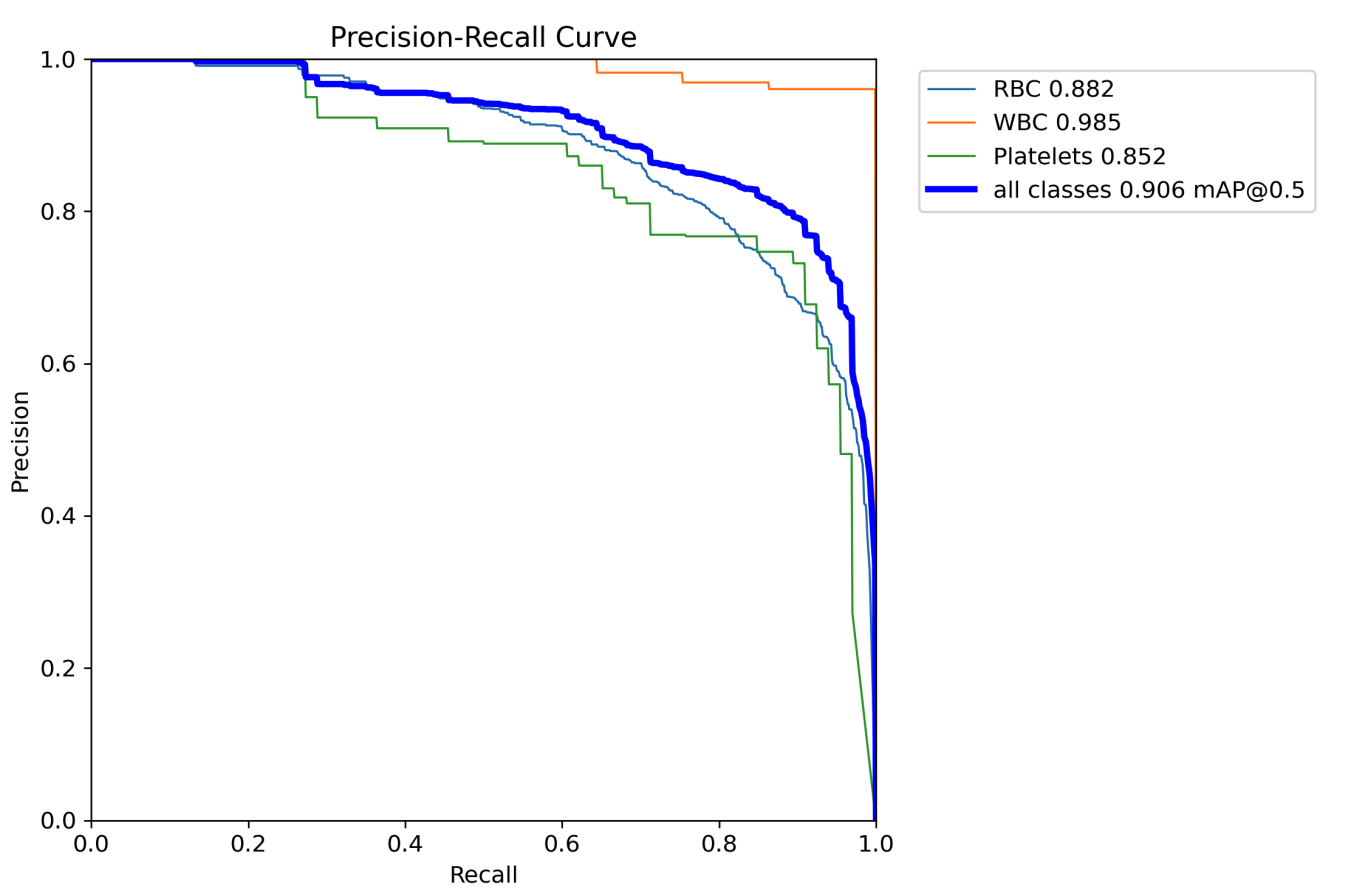
The Precision-Recall curve of YOLOv8s.

**Figure 9:**
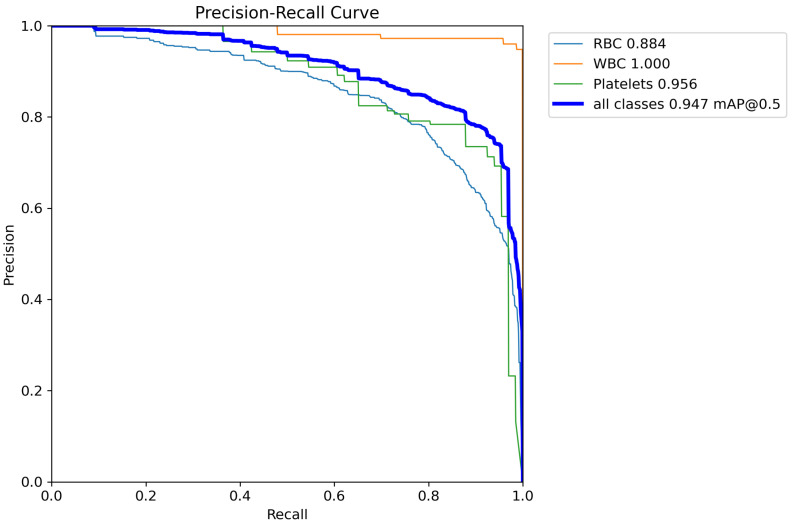
The Precision-Recall curve of TW-YOLO.

The Precision-Recall (PR) curve reflects the trade-off between precision and recall for a classification model at different thresholds. A curve closer to the top-right corner indicates better model performance, meaning it achieves higher recall while maintaining high precision. Figure 8 shows the PR curve of TW-YOLO on the BCCD dataset, while Figure 9 shows the PR curve of YOLOv8s on the BCCD dataset. From the comparison in the figures, it can be seen that TW-YOLO has a higher overall mAP50 value, which is closer to the top-right corner, indicating that TW-YOLO performs better overall in the task of blood cell detection.

**Figure 10:**
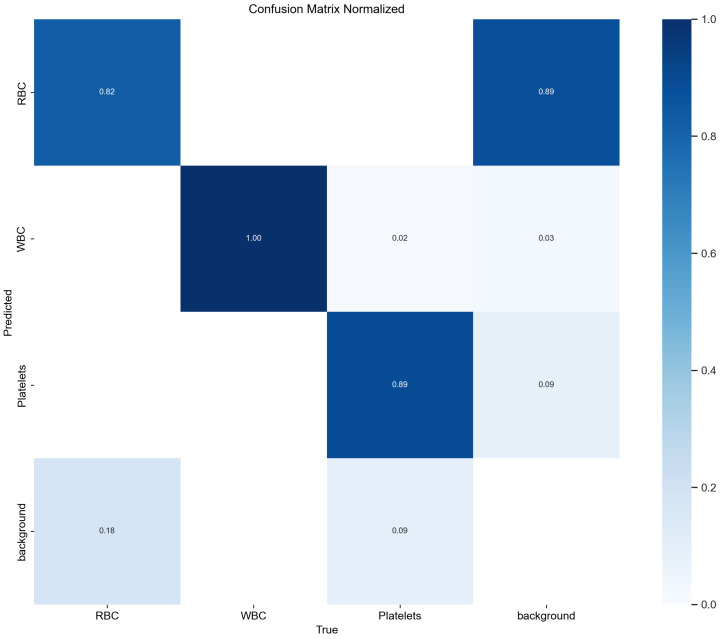
The confusion matrix of YOLOv8s.

**Figure 11:**
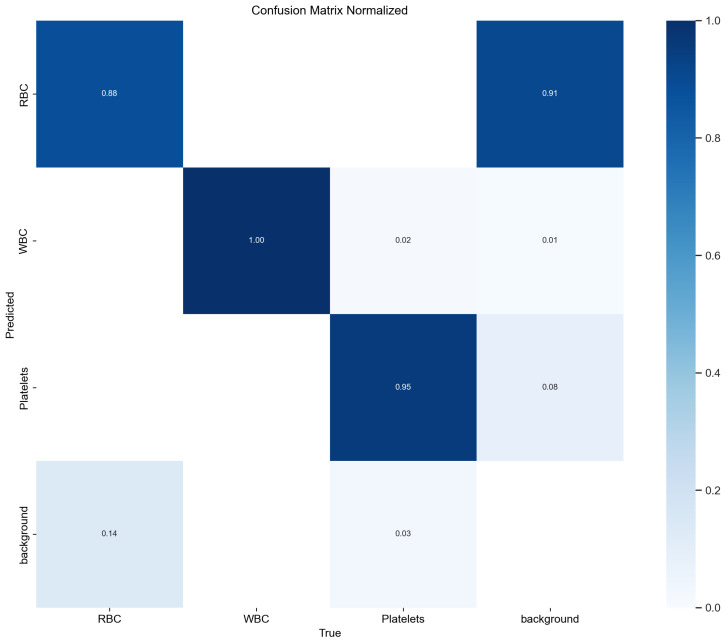
The confusion matrix of TW-YOLO.

The confusion matrix evaluates a classification model’s performance by showing the distribution of true and predicted labels across categories. Higher diagonal values and lower off-diagonal values indicate better performance with more correct classifications. Figure 10 shows TW-YOLO’s confusion matrix on the BCCD dataset, and Figure 11 shows YOLOv8s’. Comparing the figures, TW-YOLO exhibits notably better accuracy, especially for small targets, capturing features more precisely and reducing false detections. In contrast, YOLOv8s has more false positives and missed detections, while TW-YOLO consistently has higher diagonal values, reflecting improved accuracy and reliability.

**Figure 12:**
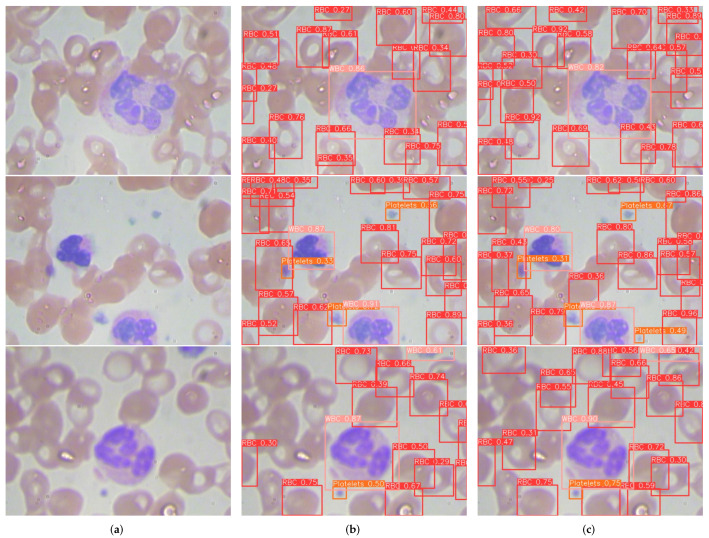
The comparison of the visualization output between YOLOv8s and TW-YOLO. (**a**) Original blood cell picture. (**b**) The YOLOv8s visualization output. (**c**) The TW-YOLO visualization output.

Figure 12 illustrates the detection visualization results comparison between YOLOv8s and TW-YOLO. TW-YOLO is able to detect blood cells at the edge of the image that YOLOv8s cannot in the first image, indicating that TW-YOLO has better detection results for blood cells of different shapes. In the second image, TW-YOLO can detect the smaller Platelets in the lower right corner, demonstrating that TW-YOLO has better detection capabilities for small-scale blood cells. In the third image, TW-YOLO shows better detection capability for overlapping blood cells. The significant improvements achieved by TW-YOLO are attributed to its sophisticated algorithm design, which can more accurately analyze the subtle differences in blood cell images, including complex color and morphological variations between cells. The model demonstrates exceptional ability in handling detailed features of blood cells with varying dimensions and shapes. The improvement in detection precision not only boosts the the model’s reliability but also significantly reduces the occurrence of false positives and false negatives.

**Figure 13:**
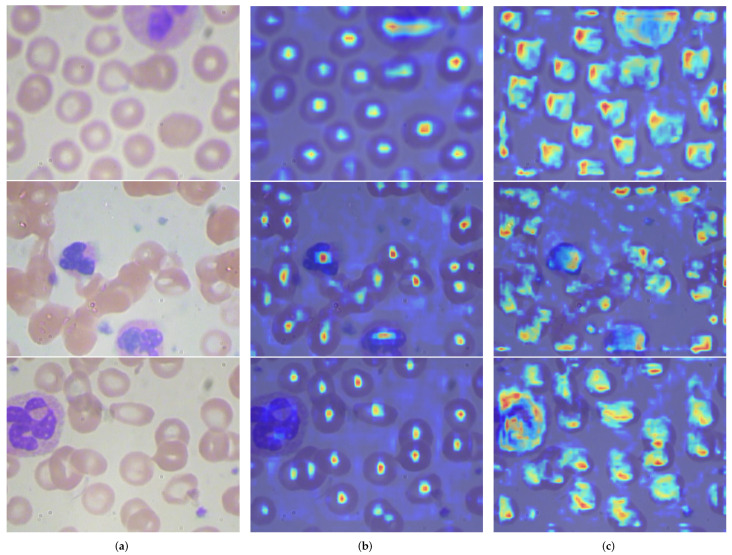
This figure shows a comparison of Grad-CAM result between YOLOv8s and TW-YOLO. The darker the color in the image, the greater the contribution of that area to the detection result. (**a**) Original blood cell picture. (**b**) The YOLOv8s Grad-CAM result. (**c**) The TW-YOLO Grad-CAM result.

We used Grad-CAM methodology [42] for feature visualization. Figure 13 shows the output of feature visualization. In the first image, we are able to see that TW-YOLO can extract more precise features based on the shape of the blood cells, indicating that TW-YOLO has better extraction capabilities for the rich geometric shapes of blood cells. In the second image, TW-YOLO can extract the smaller Platelets in the lower right corner, demonstrating that TW-YOLO has better feature extraction capabilities for small-scale blood cells. In the third image, TW-YOLO shows better feature extraction capabilities for blood cells at the edges, indicating that TW-YOLO has better feature extraction capabilities for geometric shapes. The superior feature extraction capabilities of TW-YOLO compared to YOLOv8s are attributed to the stronger feature extraction capabilities of RFAConv, EMA, and CBAM, as well as the more stable gradient flow provided by PGI-Ghost.

### 4.5. Ablation Study

We conducted ablation experiments on the BCCD dataset. Using YOLOv8s as the baseline, we gradually added each module to investigate their effects. The results of the ablation experiments are shown in Table 3. Table 3 shows the mAP50 for each category as well as the overall mAP50. By adding the EMA module, the detection accuracy improved, with all modules showing an increase, and the overall mAP50 increased by 0.7%, indicating that EMA enhances the model’s capability of extracting features for blood cells. Integrating the RFAConv module resulted in a 2.9% increase in mAP50, demonstrating that RFAConv greatly boosts the Backbone’s feature extraction ability and strengthens the model’s effectiveness in blood cell detection. By adding the PGI module, the mAP50 increased by 2%, indicating that the PGI makes the model’s gradient flow more stable. Finally, with the addition of the GhostModule, although the mAP50 decreased by about 0.5%, the number of parameters was reduced by 4M, GFLOPS decreased by about 11, and FPS increased by 39.2. This resulted in a significant speed improvement at the cost of only a minimal reduction in accuracy.

## 5. Discussion

Although the TW-YOLO model demonstrates outstanding performance in blood cell detection, there are still some limitations and future directions that deserve attention, for example, the quality and consistency of blood cell images can be affected by sample preparation, microscope settings, and shooting conditions. While TW-YOLO performs well across multiple datasets in this experiment, it is still necessary to investigate and resolve detection difficulties under extreme circumstances.

In the future, there will be multiple avenues for boosting the TW-YOLO framework. By optimizing the network structure, TW-YOLO can be made more suited for a wider range of applications, including those that demand real-time processing, by reducing computational requirements without compromising accuracy or even enhancing it. Although TW-YOLO has already reduced computational demands by incorporating the GhostModule, further research into lightweight models that do not cause substantial performance deterioration could be especially advantageous. We plan to investigate a variety of data processing methods. For instance, in cases of poor image quality, advanced image enhancement and denoising techniques can be used. In the future, our primary emphasis will be on blood cell image processing techniques to enhance object recognition in diverse circumstances. To boost the precision of the detection findings, we will refine the network by fine-tuning it to attain optimal performance.

To summarize, the TW-YOLO model has made substantial progress in blood cell detection. However, continuous efforts must be made to address its limitations. We also need to explore future development directions. This will facilitate the progress of the discipline and address the changing requirements of actual applications.

## 6. Conclusions

In this research, we introduced the TW-YOLO framework developed with the intention of blood cell detection. The model comprises five key components: the RFAConv module, which enhances feature extraction capabilities; the CBAM module, which improves feature fusion in the feature pyramid structure; the EMA module, which enhances the ability to extract target features; the GhostModule, which lowers the computing cost of the model; and the PGI-Ghost strategy, which improves the description capability of feature extraction and gradient flow in blood cell detection tasks. Our experiments on the BCCD, BCD, and CBC blood cell detection datasets demonstrate that TW-YOLO performs better than current models in identifying various blood cell kinds and in difficult background situations. Looking ahead, our goal is to continually enhance the model’s efficiency. We aim to create more efficient blood cell detection methods. This will promote the development and practical application of automated medical image analysis.

## Figures and Tables

**Table 1:** A comparison of different state-of-the-art algorithms on the blood detection dataset.

Dataset	Model	WBC	RBC	Platelets	mAP50
BCCD	YOLOv8s [35]	1	0.822	0.894	0.905
	YOLOv5x [36]	0.977	0.877	0.915	0.923
	YOLOv7 [37]	0.977	0.829	0.883	0.896
	CST-YOLO [38]	0.984	0.869	0.928	0.927
	YOLOv9s [29]	0.984	0.834	0.903	0.907
	YOLOv10s [39]	0.984	0.842	0.912	0.913
	TW-YOLO	**1**	**0.884**	**0.956**	**0.947**
BCD	YOLOv8s [35]	1	0.912	0.924	0.945
	YOLOv5x [36]	0.995	0.930	**0.942**	0.955
	YOLOv7 [37]	0.995	0.917	0.912	0.941
	CST-YOLO [38]	0.995	0.947	0.927	0.956
	YOLOv9s [29]	0.995	0.921	0.927	0.948
	YOLOv10s [39]	1	0.930	0.932	0.954
	TW-YOLO	**1**	**0.954**	0.932	**0.962**
CBC	YOLOv8s [35]	1	0.914	0.926	0.947
	YOLOv5x [36]	0.820	0.857	0.975	0.884
	YOLOv7 [37]	0.874	0.785	0.974	0.878
	CST-YOLO [38]	0.899	0.857	**0.978**	0.911
	YOLOv9s [29]	0.932	0.921	0.946	0.933
	YOLOv10s [39]	0.956	0.937	0.926	0.940
	TW-YOLO	**1**	**0.955**	0.946	**0.967**

**Table 2:** A comparison of different state-of-the-art algorithms on LISC detection dataset.

Model	NEU	EOS	MON	BAS	LYM	mAP50
YOLOv8s	0.79	**0.835**	0.802	0.637	0.771	0.980
DETR [40]	0.821	0.760	0.806	**0.777**	0.725	0.989
YOLOv5-ALT [41]	**0.843**	0.745	**0.841**	0.595	0.771	0.988
YOLOv9s [29]	0.791	0.767	0.794	0.647	0.763	0.974
YOLOv10s [39]	0.810	0.791	0.804	0.657	0.756	0.984
TW-YOLO	0.831	0.817	0.734	0.679	**0.799**	**0.993**

**Table 3:** The result of the ablation study on the BCCD dataset.

Model	WBC	RBC	Platelets	mAP50	Parameter	GFLOPs	FPS
YOLOv8s	1	0.822	0.894	0.904	11.1M	28.6	186.8
+EMA	1	0.831	0.902	0.911	11.4M	31.2	156.4
+CBAM	1	0.840	0.913	0.918	11.9M	33.4	134.5
+RFAConv	1	0.865	0.931	0.932	13.9M	45.4	96.3
+PGI	1	0.895	0.961	0.952	17.1M	49.6	65.1
+GhostModule	1	0.884	0.956	0.947	13.0M	38.6	104.3

## Data Availability

All datasets are publicly available, and the acquisition methods are provided in the article and in the links within the references.
